# Report of Special Committee on Dental Nomenclature

**Published:** 1895-12

**Authors:** S. H. Guilford

**Affiliations:** Philadelphia, Pa.


### Report of Special Committee on Dental Nomenclature.

BY S. H. GUILFORD, D.D.S., PHILADELPHIA, PA.

Abstract of report read at the American Dental Association, Aug., 1895.

The nomenclature of any science or art, like a language, is not a creation, but a growth. It starts with a small beginning, and is developed gradually.

Frequently the growth of a science or art is so rapid that the terms selected to convey the necessary ideas are chosen without a proper regard to their fitness. Such has been the case with many sciences, and such has, unfortunately, been the case with our own.

We must move slowly and carefully, and advance only as rapidly as we succeed in convincing our fellows of the advisability of the change. Terms that are scientifically incorrect should be discarded at once and correct ones substituted; those that are not absolutely incorrect may be tolerated for awhile longer, but for the new ones which must necessarily be introduced from time to time, this association should establish a standard or code and use its great influence in securing its approval and adoption by the profession in this country at least.

While the establishment of a system of nomenclature of international character would be most desirable, the present does not appear to be the time in which to attempt it, but if we can devise and adopt a system for our own country that has a scientific basis and is well adapted to our wants, we believe that it will eventually be adopted in whole or in part by other nations. The framing of a code is a task of considerable difficulty, for no one language contains all of the elements from which to construct a perfectly correct and convenient nomenclature. A living language will not do, because it is constantly changing. The choice, therefore, would seem to lie between one of the two dead languages, Latin or Greek.

In the science of electricity, and indeed physics in general, in geology and mineralogy, in theology, in medicine and surgery—especially in the departments of bacteriology and pathology—almost the entire list of recent terms is constructed from Greek roots. If, therefore, these sister sciences have deemed it best and most satisfactory to base their newer terminology upon a Greek foundation, can we do better than follow their example and keep in scientific accord?

In case we decide to accept the Greek language as a basis for our terminology, however, it does not follow that we must adhere to it rigidly in all cases, for in many instances, no doubt, the Latin, English, or other languages will furnish us with terms that are equally as good and probably simpler in form than corresponding ones derived from the Greek.

Very many of our present terms have a Latin derivation, having been borrowed from our sister profession, medicine; and where these have proven satisfactory and are not etymologically incorrect, we may allow them to remain. Many also have their foundation in Greek, as "gypsum," "asbestos," "prosthesis," "technics," etc.: while a few, like "celluloid," "centimeter," etc., are half Latin and half Greek. In the descriptive anatomy of the teeth, your committee is well pleased with the plan and terms proposed by Dr. Black two years ago, but some modification of that plan will probably be necessary.

Inasmuch as the terms used by common consent to indicate four of the surfaces of the crown of the tooth end in the Latin adjective terminal "al," your committee favors the adoption of a word having the same ending for the fifth or antagonizing surface. Whether this shall be "occlusal" for the bicuspids and molars, and "incisal" for the incisors, as suggested by Dr. Black, or "morsal," for all, as proposed by Dr. Kirk, remains to be determined. Your committee believes that the use of the single term "occlusal" for this surface of all of the teeth will serve our purpose best. As between the words "gingival" and cervical," we recommend the adoption of the latter, because it is exact in its meaning, and has the sanction of long use.

In naming the teeth themselves we strongly condemn the use of such unscientific terms as "sixth-year molar," "twelfth-year molar," and "wisdom-tooth," and urge instead designating them first, second, and third molars respectively.

We also believe it to be more harmonious and appropriate to use the word "cuspid" instead of "canine" to indicate the single-cuspid teeth, just as the term "bicuspid" is applied to their adjoining neighbors. So, also, in indicating a particular tooth, it is more methodical and correct to proceed from the general to the particular, naming first the jaw, then the side, and lastly the tooth, as: "superior, left, second molar."

The elision of the final vowel in such words as dentin, iodin, etc., as approved by such authorities as the Century and Standard dictionaries, your committee heartily concurs in and recommends.

Your committee would beg to remind the association that this report marks but the beginning of its labors, and that much time and effort will be required to obtain anything like a complete or satisfactory result.

